# Bereavement Support Services in a National Sample of Hospices: A Content Analysis

**DOI:** 10.1093/geroni/igab046.2186

**Published:** 2021-12-17

**Authors:** Todd Becker, John Cagle

**Affiliations:** University of Maryland, Baltimore, Baltimore, Maryland, United States

## Abstract

Although the Medicare Hospice Benefit mandates that hospices provide bereavement services to families for 1 year following death, it does not stipulate what services should be offered or how. Thus, this study aimed to explore the range of hospice bereavement services. This study stems from Cagle et al.’s (2020) prior study surveying 600 randomly selected agencies, stratified by state and profit status. Most participants (N = 76) worked as clinical supervisors or directors of patient services (41.6%) for medium-sized (53.2%), for-profit hospices (50.6%). Responses to “What types of bereavement support does your hospice provide to families?” were content analyzed. Analyst triangulation and peer debriefing enhanced trustworthiness. Four domains emerged: timing of support, providers of support, targets of support, and formats of support. Each domain reflected substantial variability. All hospices offered postdeath bereavement support. A minority described offering predeath support, often through bereavement risk assessment and supportive services targeting those at risk. Providers frequently included trained bereavement counselors, social workers, and chaplains. Less often, hospices leveraged familiar members of the decedents’ care team to encourage family participation. Although bereavement services predominantly targeted surviving adult family members of deceased hospice patients, services tailored to children and hospice-unaffiliated community members also emerged. The format of bereavement services demonstrated the widest variability. Commonly reported formats included written materials, support groups, and phone calls. Most hospices employed multiple formats. Although findings are consistent with prior research, the variability in each domain complicates rigorous investigation of which aspects offer the greatest benefit to bereaved family members.

